# Supplementary material for: Synergistic Anticancer Activity of HSP70 Inhibitor and Doxorubicin in Gain-of-Function Mutated p53 Breast Cancer Cells
Source: Biomedicines. 2025 Apr 24;13(5):1034. doi: 10.3390/biomedicines13051034 (PMC12109493; doi:10.3390/biomedicines13051034)
Supplement: Supplementary file 1 [file biomedicines-13-01034-s001.zip › biomedicines-3560017-supplementary.pdf]

Supplemental Information

Synergistic anticancer activity of HSP70 inhibitor and doxorubicin in gain-of-function mutated p53 breast cancer cells

Kuan-Yo Wu, Ana Crucho, Mia Su, Sih-Tong Chen, Chen-Hsiu Hung, Yu-Ling Kou, Yu-Jie Liu, Tzu-Chi Hsu, Fang-Yu Yeh, Ching-Feng Lien, Chia-Chi Chen and Bi-He Cai

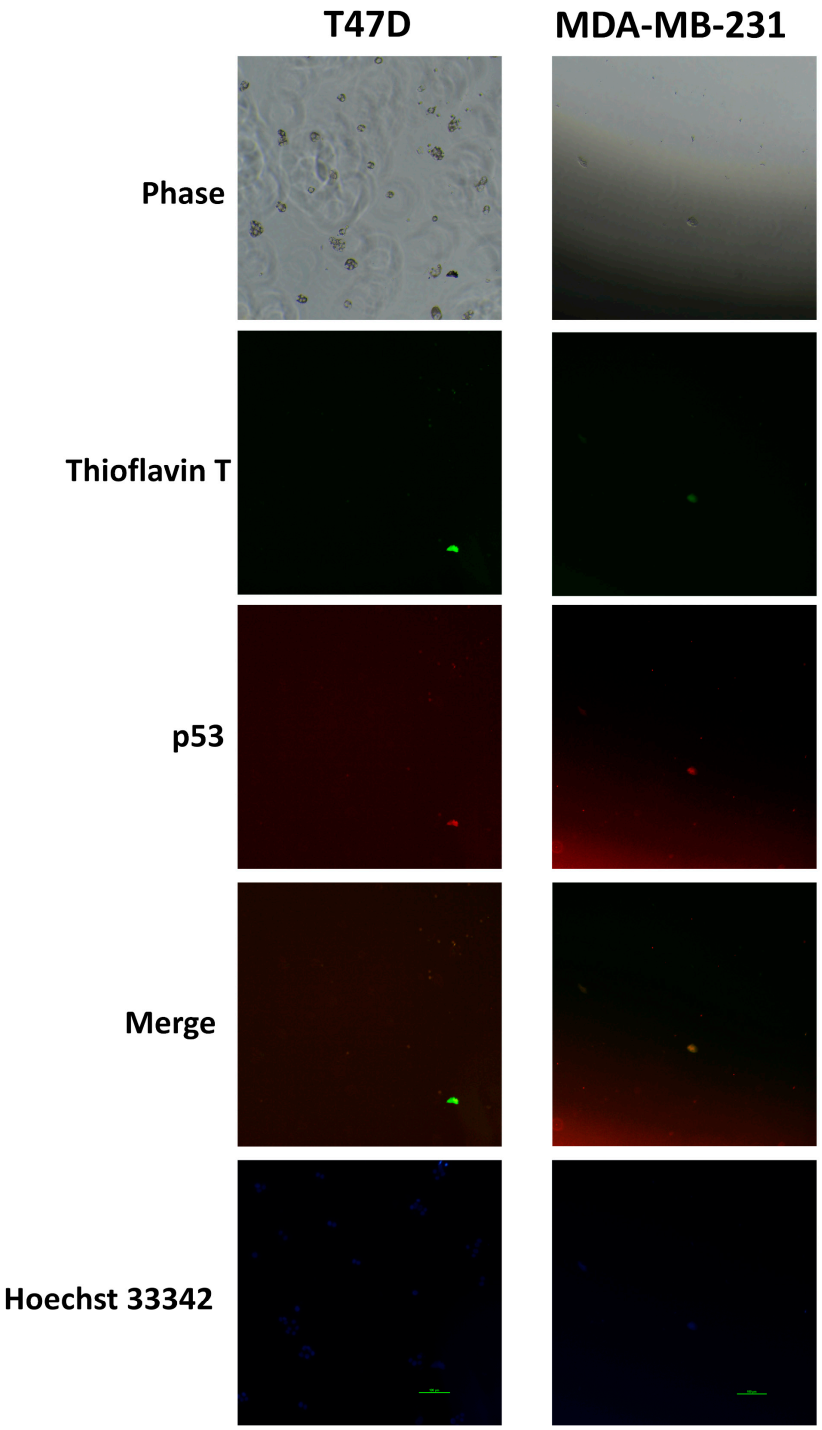

Figure S1. p53 colocalizes with thioflavin T in T47D and MDA-MB-231 cells. was used to stain protein aggregates (shown in green); p53 is shown in red. Hoechst 33342 was used to stain the nuclei of cells (shown in blue). The merged image shows that the p53 signals overlap with the thioflavin T signals. Scale bar: 100 µm (20× magnification).

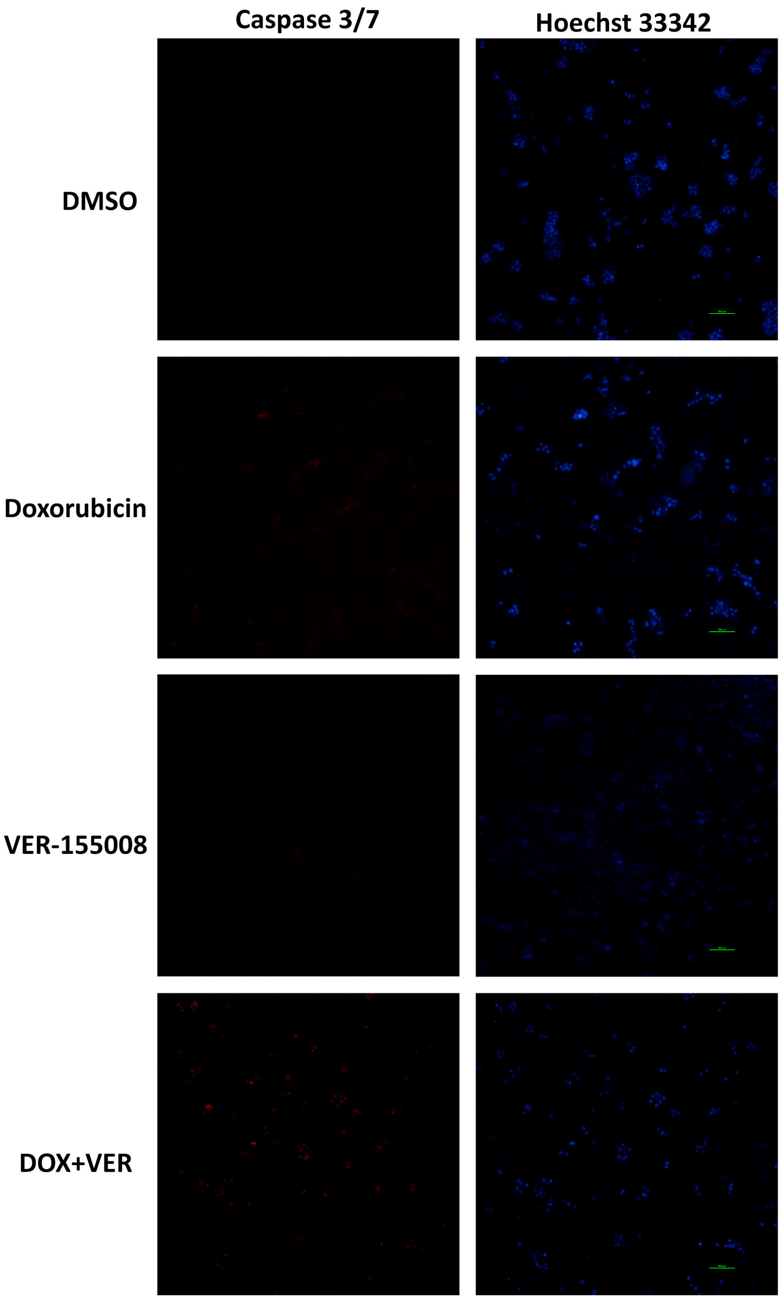

Figure S2. Detection of activated caspase-3/7 following drug treatment in T47D cells. Activated caspase-3/7 (red) is clearly induced by 24-hour co-treatment with doxorubicin (1 µM) and VER-155008 (10 µM) in T47D cells. Hoechst 33342 was used for nuclear counterstaining (shown in blue). Scale bar: 100 µm (20× magnification).

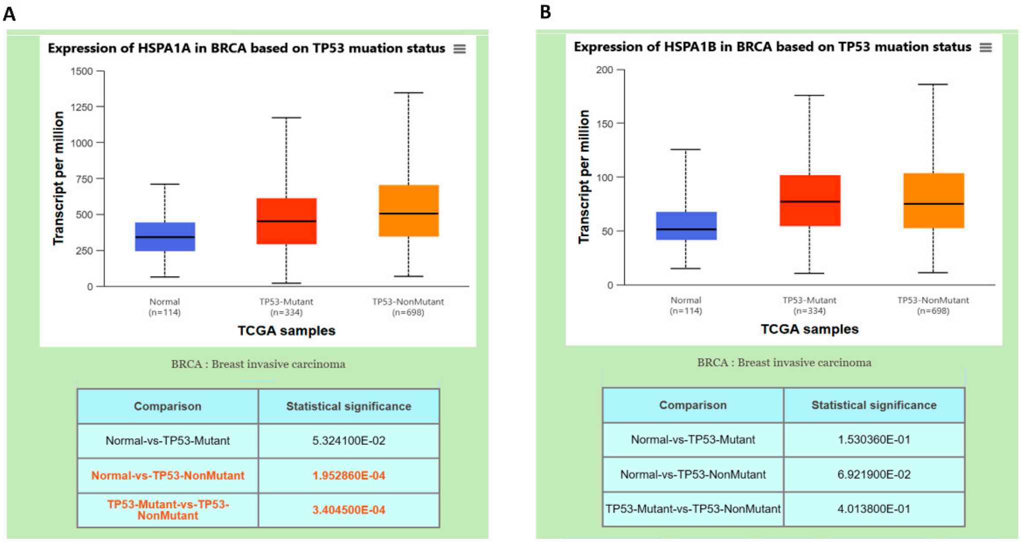

Figure S3. Expression profiles of the Hsp70 family members HSPA1A and HSPA1B in breast invasive carcinoma (BRCA) bearing wild-type or mutated p53. (A) According to the UALCAN database (https://ualcan.path.uab.edu/cgi-bin/TCGAExResultNew2.pl?genenam=HSPA1A&ctype=BRCA [accessed on 3 April 2025]), HSPA1A was highly expressed in both wild-type and mutated p53 BRCA compared to healthy tissue, but HSPA1A expression showed relatively lower expression in p53-mutated BRCA compared to BRCA with wild-type p53 (B) According to the UALCAN database (https://ualcan.path.uab.edu/cgi-bin/TCGAExResultNew2.pl?genenam=HSPA1B&ctype=BRCA [accessed on 3 April 2025]), HSPA1B was highly expressed in both wild-type and p53-mutated BRCA compared to healthy tissue.

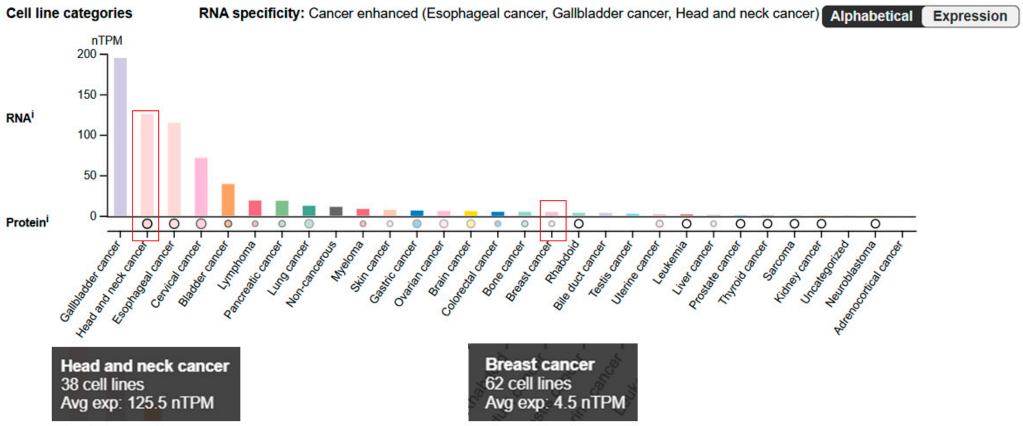

Figure S4. The p63 expression pattern in different cancer cell lines. According to the RNA sequencing database of cell lines in the Human Protein Atlas (https://www.proteinatlas.org/ENSG00000073282-TP63/cell+line [accessed on 1 March 2024]), the average value of nTPM of p63 in thirty-eight different head and neck cancer cell lines is 125.5. The average nTPM value of p63 in sixty-two different breast cancer cell lines is only 4.5.

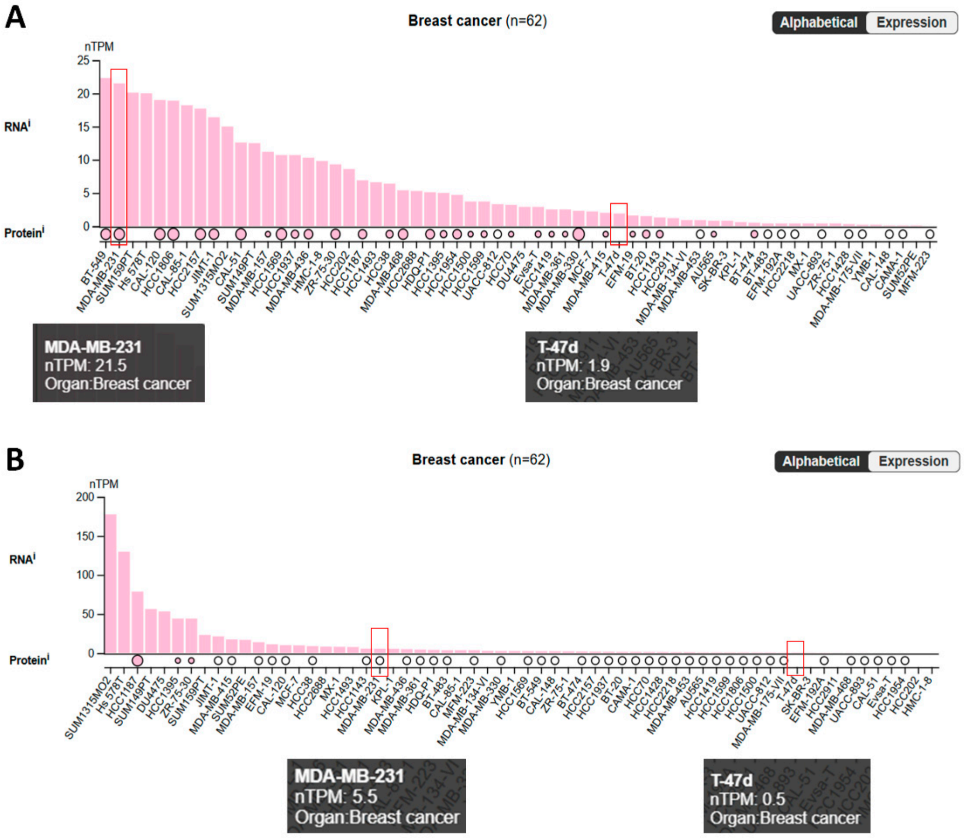

Figure S5. The expression patterns of BAG2 and LOXL1 in different breast cancer cell lines. (A) According to the RNA sequencing database of cell lines in the Human Protein Atlas (https://www.proteinatlas.org/ENSG00000112208-BAG2/cell+line [accessed on 1 March 2024]), the nTPM of BAG2 is 21.5 in MDA-MB-231 cells, but the nTPM of T47D is only 1.9. (B) According to the RNA sequencing database of cell lines in the Human Protein Atlas (https://www.proteinatlas.org/ENSG00000129038-LOXL1/cell+line [accessed on 1 March 2024]), the nTPM of LOXL1 is 5.5 in MDA-MB-231 cells, but the nTPM of T47D is only 0.5
